# Sustainable Membrane Technologies for Enhancing Urban Climate Resilience

**DOI:** 10.3390/membranes16020070

**Published:** 2026-02-19

**Authors:** Andreea Loredana Rhazzali, Elena Simina Lakatos, Ráhel Portik-Szabó, Elena Cristina Hossu, Lucian-Ionel Cioca, Alina Moldovan

**Affiliations:** 1 Institute for Research in Circular Economy and Environment Ernest Lupan, 400561 Cluj-Napoca, Romania; loredana.rhazzali@ircem.ro (A.L.R.); simina.lakatos@ircem.ro (E.S.L.); rahel.portikszabo@ircem.ro (R.P.-S.); cristina.hossu@ircem.ro (E.C.H.); lucian.cioca@ulbsibiu.ro (L.-I.C.); 2 Academy of Romanian Scientists, 3 Ilfov Street, 050044 Bucharest, Romania; 3 Department of Economics, Faculty of Horticulture and Rural Development Business, University of Agricultural Sciences and Veterinary Medicine, 400372 Cluj-Napoca, Romania; 4 Faculty of Engineering, Lucian Blaga University of Sibiu, 550024 Sibiu, Romania

**Keywords:** membrane technology, urban climate resilience, wastewater, reuse, cooling, nature-based solutions

## Abstract

Growing wastewater volumes and intensifying water scarcity are driving the need for affordable, sustainable solutions that enable safe urban water reuse and strengthen climate resilience. Policy frameworks such as SDG6 and EU water reuse requirements highlight that reclaimed water must meet strict environmental and public health standards. In contrast, conventional biological treatment cannot fully remove many emerging contaminants, underscoring the need for advanced treatment approaches that consistently deliver high-quality reclaimed water. In this context, this review examines the role of membrane technologies (MF, UF, NF, RO, FO) and membrane bioreactors (MBRs) in providing safe water in urban environments and in enhancing urban climate resilience, including decentralized systems and advanced reclamation needs. It also discusses the contribution of membrane-based solutions to sustainable cooling systems and heat-stress mitigation, as well as the integration of membrane technologies into green infrastructure and nature-based solutions for climate adaptation. Technical and economic performance is shaped by fouling, cleaning requirements, and energy use, making life-cycle and operational optimization critical for long-term sustainability. Case studies and EU-funded initiatives demonstrate that, with appropriate governance and design, membrane-based approaches can enable reliable reclaimed water supply, enhance water security, and contribute to circular urban water management. The analysis was based on peer-reviewed open-access publications, which may introduce a degree of selection bias.

## 1. Introduction

Water scarcity is a global challenge intensified by pollution and poor resource management [1]. At the same time, global population growth and expanding industrial activities have substantially increased wastewater generation [2]. Reuse of low-quality water sources, including wastewater, urban groundwater, and stormwater, can help alleviate freshwater shortages; however, safe application requires effective treatment to remove chemical and biological contaminants [3,4]. Urban areas represent the most densely populated artificial environments, and their role in global population distribution continues to expand, with projections indicating that more than 60% of the world’s population will live in cities by 2030. Environmental changes associated with urbanization frequently lead to environmental degradation and increased risks to human health [5].

Rapid urbanization, climate change, and increasing pressure on natural resources have made sustainable urban water management an urgent priority. As cities account for the highest levels of water consumption, ensuring a reliable and sustainable water supply requires integrated management approaches across the entire urban water cycle. The adoption of advanced technologies plays a crucial role in improving water-use efficiency, promoting responsible consumption patterns, and maintaining water quality within complex urban ecosystems [6].

For reuse applications that do not require high-quality water, such as irrigation of non-food crops or urban cleaning, conventional treatment processes, including coagulation, sedimentation, filtration, biological treatment, and disinfection, are often sufficient [7]. In contrast, applications requiring high-quality reclaimed water, such as industrial and potable reuse, necessitate advanced membrane technologies, as demonstrated by techno-economic analyses comparing energy consumption and performance across different reuse types [8].

Research indicates that nature-based solutions (NBS) can contribute to social innovation in cities and accelerate the transition toward sustainability [9]. These solutions are increasingly used to restore ecological processes in urban environments while providing new infrastructure pathways to enhance urban resilience. In recent years, growing interest from urban planners and policymakers has increased the demand for systematic assessment and scientific evidence of the multiple benefits of nature-based solutions [10].

International and European policy frameworks strongly support safe water reuse. The United Nations Sustainable Development Goal 6 emphasizes integrated water resources management and the global implementation of safe water reuse by 2030 [11]. Similarly, the EU Water Reuse Regulation defines minimum quality requirements to protect public health and the environment while promoting circular economy practices [12]. These frameworks highlight that conventional biological treatment processes alone are insufficient to remove many contaminants of emerging concern, such as pharmaceuticals, hormones, pesticides, and personal-care products, which persist in treated wastewater [13]. This limitation reinforces the need for advanced treatment solutions, including membrane-based systems and membrane bioreactors, which demonstrate high removal efficiencies for nutrients, organic matter, pathogens, and micropollutants [14].

Globally, climate change, population growth, and inefficient water management have left approximately 1.1 billion people without access to safe drinking water, while 2.5 billion lack adequate sanitation, including an estimated 41 million people in Europe alone. These challenges have accelerated the adoption of membrane technologies, driven by declining water quality, stricter regulations, growing demand for high-quality potable water, and the increasing presence of hazardous environmental pollutants [15]. Advanced membrane technologies therefore represent a key contribution to addressing water insecurity and enhancing sustainability by supporting water purification, ecosystem protection, sustainable urban development, and circular economy objectives [16,17].

To address this issue, this paper conducts a systematic review of sustainable membrane technologies for climate resilience in the urban environment. To guide this investigation and ensure a structured and coherent analysis, the following research questions have been formulated:

1. How do membrane technologies contribute to enhancing urban climate resilience?
2. How are membrane filtration systems applied in water reuse, decentralized treatment, and stormwater management?
3. What role do membrane-based solutions play in sustainable cooling systems and heat-stress mitigation?
4. How can membrane technologies be integrated into green infrastructure and nature-based solutions for climate adaptation?

## 2. Methodology of the Research: Data Sources and Analysis Methods

To obtain a comprehensive and well-structured understanding of how membrane technologies contribute to urban climate resilience, a systematic literature review was conducted (The PRISMA checklist is included in Supplementary Materials). The methodological approach was primarily based on querying the Web of Science database, which represents one of the most comprehensive sources for sustainability-related research. The initial search employed a topic-based query, covering titles, abstracts, and author keywords, using the combined search terms “membrane” AND “urban”. This search yielded a total of 2165 records.

**Figure 1:**
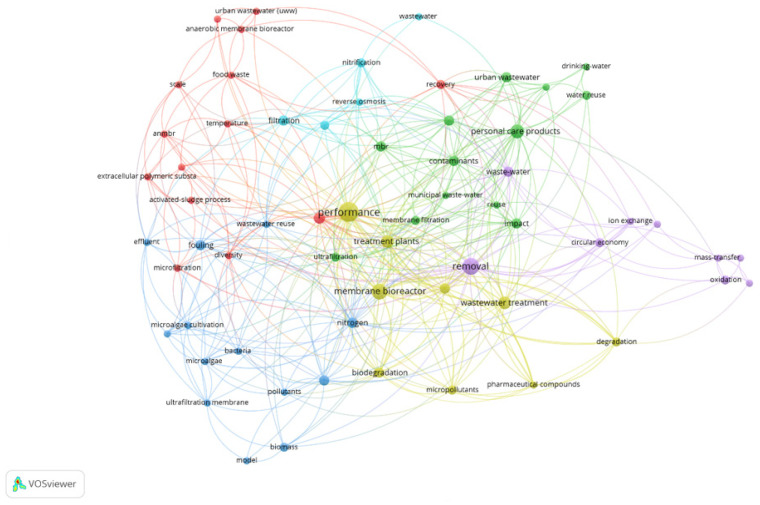
Network visualization of keyword co-occurrence related to membrane technologies in urban contexts. The network highlights key research themes, their interconnections, and the dominant thematic clusters guiding the subsequent content analysis.

Subsequently, the retrieved publications were narrowed down by applying several filtering criteria, including publication year (2000–2025), language (English), access type (open access), and document type (peer-reviewed articles and review papers). Following a screening of publication titles and the selection of studies relevant to the research scope, 758 articles were retained and imported into VOSviewer (version 1.6.20) for keyword co-occurrence analysis. A minimum keyword occurrence threshold of 2 was applied, resulting in 59 keywords meeting the threshold out of 434 identified keywords (Figure 1).

**Figure 2:**
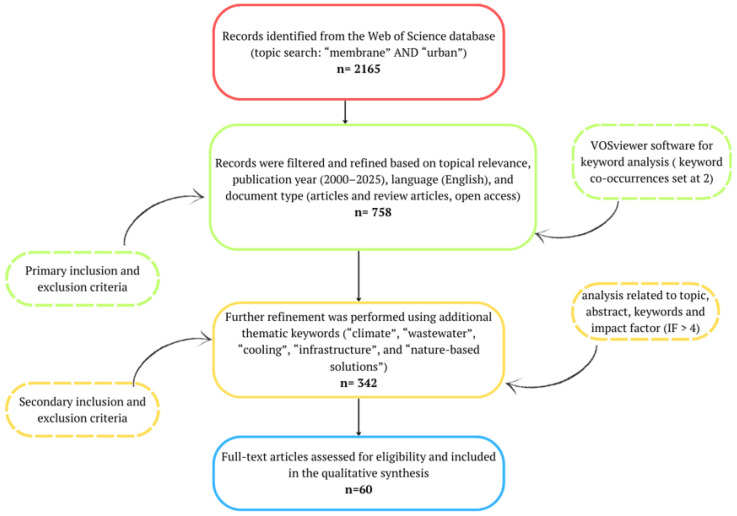
Research methodology based on the Web of Science database for analyzing the role of membrane technologies in enhancing urban climate resilience.

To align the dataset more explicitly with the objectives of urban climate resilience, the initial search strategy (“membrane AND urban”) was refined by adding thematic keywords related to “climate”, “wastewater”, “cooling”, “infrastructure”, and “nature-based solutions”. This refinement resulted in 342 publications, which were examined in relation to the role of membrane technologies in enhancing urban climate resilience. Based on an in-depth relevance assessment of abstracts and keywords, as well as journal impact factors (IF > 4), 60 articles were ultimately selected for detailed qualitative analysis aligned with the objectives of this study (Figure 2).

Following a detailed analysis of the selected literature, the relevant information was organized within a contextual and content analysis framework, with a specific focus on examining the role of membrane technologies in urban climate adaptation. The context analysis explored the spatial and temporal patterns reflecting the diffusion and evolution of membrane-based solutions in urban environments. In parallel, the content analysis concentrated on technological developments, key application areas, namely water reuse, cooling systems, and green infrastructure, as well as the identified challenges and opportunities for further improvement, while also considering their alignment with urban climate adaptation strategies and European policy frameworks (Figure 2).

## 3. Results

### 3.1. Context Analysis

Urban climate adaptation and resilience have become increasingly prominent topics in the scientific literature, particularly regarding the application of membrane technologies in urban environments. The reviewed studies provide a comprehensive overview of how membrane-based solutions enhance the adaptive capacity of urban systems in response to challenges such as heat waves, water scarcity, increased rainfall intensity, and declining urban water quality. At the same time, the literature highlights a range of limitations and structural barriers that currently hinder the widespread and integrated implementation of these technologies in urban contexts.

**Figure 3:**
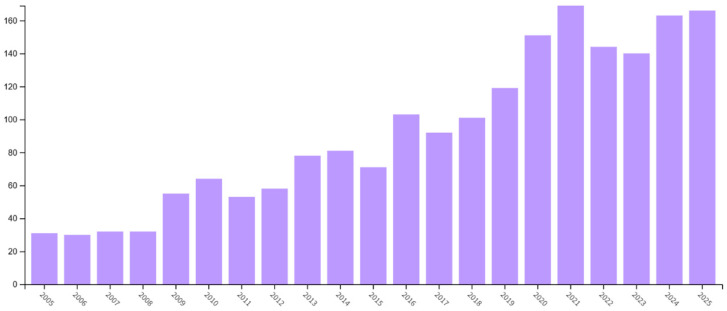
Temporal evolution of publications indexed in the Web of Science database focusing on the relationship between membrane technologies and urban environments.

The temporal dimension of the context analysis indicates a marked increase in research activity addressing the relationship between membrane technologies and urban climate adaptation, especially over the past decade. Publication trends reveal substantial growth in the number of studies, reflecting rising scientific and practical interest in membrane-based solutions for strengthening urban climate resilience (Figure 3).

**Figure 4:**
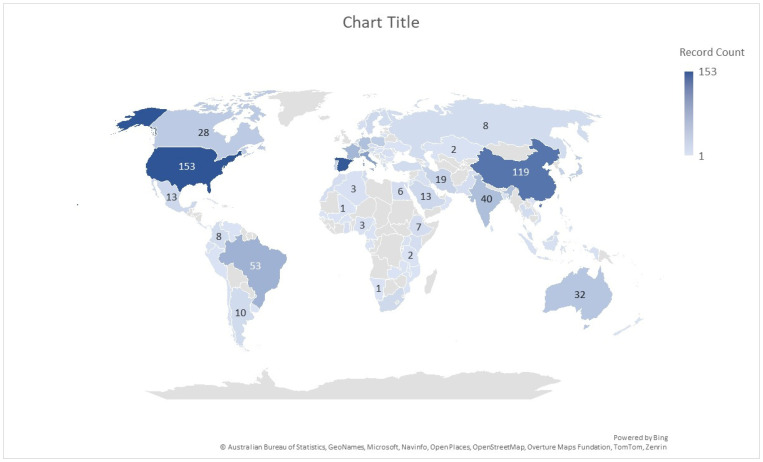
Spatial distribution of publications indexed in the Web of Science database addressing membrane technologies in urban contexts.

The spatial component of the context analysis (Figure 4) demonstrates a broad geographical distribution of research efforts, suggesting that the urban application of membrane technologies has emerged as a topic of global relevance. A high concentration of publications originates from Europe, America, and Asia, which corresponds to significant urbanisation pressures, climate-related risks, and increasing demand for sustainable urban water and energy systems in these regions. This geographical diversity reflects ongoing efforts to integrate membrane technologies into urban climate adaptation strategies, including applications in water reuse, cooling systems, and green infrastructure.

### 3.2. Content Analysis

Building on the spatial and temporal patterns identified in the context analysis, the content analysis synthesises evidence from the selected literature and is structured around the main thematic areas of the review. It examines how membrane technologies are discussed and applied in relation to urban climate resilience, with particular emphasis on (i) urban climate challenges and adaptation needs, (ii) water reuse and decentralised treatment, (iii) membrane-based cooling systems for heat mitigation, and (iv) integration into green infrastructure and nature-based solutions. Within these themes, the analysis reviews key membrane technologies (MF, UF, NF, RO, FO, and MBRs), their application contexts, and recurring performance- and operation-related factors affecting feasibility and long-term implementation. In line with the review objectives, it also highlights commonly reported constraints and enabling conditions, particularly those related to fouling and cleaning requirements, energy use, and economic and governance aspects, where explicitly addressed in the literature.

#### 3.2.1. Urban Climate Challenges and Adaptation Needs

The increasing frequency and intensity of weather extremes are reshaping urban climates and increasing cities’ exposure to hazards such as heatwaves, heavy rainfall, flooding, storms, and droughts [18]. Urbanization amplifies these risks by altering atmospheric conditions through built structures, emissions, and land-use changes, leading to phenomena such as the urban heat island, modified wind patterns, and disrupted surface energy balances. These human-induced modifications influence temperature, humidity, and airflow, thereby defining the conditions under which extreme climate events occur in cities [19].

The urban heat island is one of the most significant urban climate impacts, characterized by persistently higher air temperatures than the surrounding rural areas. Its intensity increases with the degree of urbanization, particularly in rapidly developing cities where vegetation loss, land cover change, and anthropogenic heat emissions contribute to warming. Consequently, extreme heat events become more frequent, energy demand rises, air quality deteriorates, and health risks increase, making the urban heat island a critical driver of urban vulnerability and adaptation needs [20]. Densely built surfaces further intensify heat retention, increasing pressure on cooling demand, public health, and infrastructure resilience [21].

**Figure 5:**
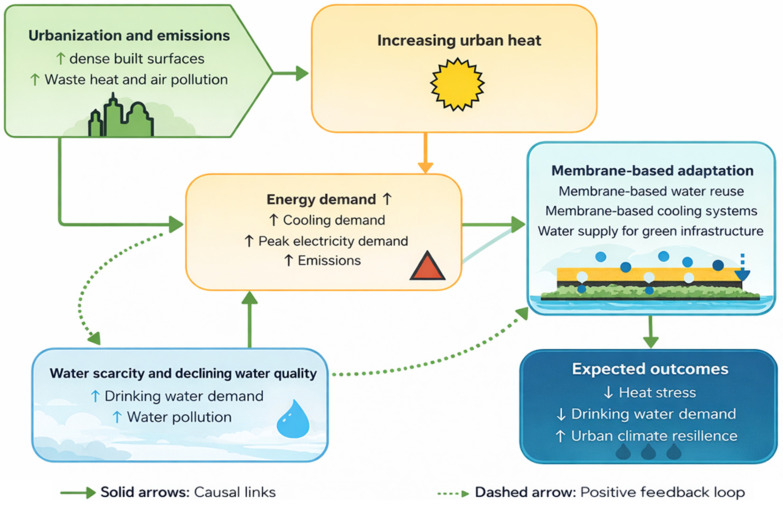
Conceptual illustration of major urban climate challenges driving adaptation needs.

Long-term observations indicate that urban heat island intensity has increased by approximately 0.021 °C per year, a trend that may generate additional thermal stress comparable to projected global warming by the end of the century. Rising nighttime temperatures limit heat release from buildings, exacerbate summertime overheating, and increase energy demand, while reduced wind speeds, lower cloud cover, and vegetation loss further weaken natural cooling mechanisms (Figure 5). These trends underscore the urgent need for urban adaptation strategies that mitigate heat stress and enhance climate resilience [22].

Water scarcity and declining water quality represent parallel challenges for urban climate adaptation, both intensified by urbanization and climate change [23]. In many regions, the safe reuse of urban wastewater has become essential, yet dissolved organic matter (DOM) remaining after secondary treatment poses a significant barrier to reuse [24]. Advanced membrane technologies (UF, RO) are therefore required to reduce risks and improve water security [25].

In Europe, water stress and water quality degradation have intensified, with nearly half of European countries—representing around 70% of the population—already affected. Water stress is particularly pronounced in semi-arid coastal regions and densely urbanized areas and is expected to worsen under changing climatic conditions. These pressures challenge municipalities facing rising demand, higher supply costs, and competition among agriculture, industry, and tourism, reinforcing the need for integrated water management approaches promoted by the Water Framework Directive [26].

#### 3.2.2. Membrane Technologies for Water Reuse

##### Types of Membrane Technologies and Their Applications

This section does not aim to provide a general overview of membrane technologies, which is already well covered in the literature, but rather to synthesize their applications specifically in the context of urban climate resilience. The focus is placed on how different membrane technologies support water reuse, decentralized treatment, and water–energy interactions relevant to urban adaptation strategies.

Membrane-based processes such as RO (reverse osmosis) and NF (nanofiltration) are widely used in wastewater treatment to meet the increasing demand for clean water. Their broad application is driven by the fact that membrane technologies are simple to operate, cost-effective, compact, and capable of delivering high performance [27].

RO uses a semi-permeable membrane with pores smaller than 1 nm to separate water from dissolved minerals and organic substances. During the process, pressure is applied to push the solvent through the membrane toward a cleaner water stream. RO is best known for producing drinking water by removing salt and other impurities. Today, it is widely used around the world for industrial, residential, commercial, and scientific water treatment at relatively low cost [28].

NF membranes with high water permeance and strong rejection capability are well-suited for desalination and wastewater treatment [29]. Typical NF membranes used in desalination consist of a porous support layer and a thin polyamide selective layer. Compared to RO membranes, NF membranes are applied where partial softening or selective removal of organic compounds, color, or specific monovalent ions is required, such as Li/Mg separation in brine treatment [30]. They are therefore mainly used for brackish water treatment or industrial processes where full desalination is unnecessary [31]. Nanofiltration membranes enable efficient removal of microscopic contaminants (0.001–0.01 μm) from wastewater and have shown high effectiveness in eliminating micropollutants and dyes from printing and textile dyeing effluents. Compared with UF membranes, NF membranes achieve higher rejection rates while operating at lower pressures and higher permeate fluxes than RO membranes [32]. However, their selectivity may decline in applications requiring dye–salt separation, prompting the development of hydrophilic, loose NF membranes with pore sizes between those of NF and UF membranes [33]. NF membranes are also applied as tertiary treatment in wastewater treatment plants to remove salts and micropollutants. However, their rejection of sodium, chloride, and nitrate ions remains lower than that of RO membranes [34].

NF also faces challenges, as the molecular weights of many micropollutants, such as pharmaceuticals, hormones, and pesticides, are close to the molecular weight cut-off of NF membranes (≈200–1000 Da); therefore, their removal does not depend solely on size, but also on molecular charge, pH-dependent ionization, hydrophobic/hydrophilic properties, and the surface charge of the membrane [35].

Microfiltration (MF) and ultrafiltration (UF) membranes are among the most advanced membrane technologies used in wastewater treatment. Both processes are pressure-driven, but they differ in membrane pore size: MF membranes typically range from 0.1 to 5 μm, while UF membranes range from 0.01 to 0.1 μm [36].

A wide range of polymer-based MF membranes has been tested for various wastewater streams, including oily effluents, runoff water, and wastewater from dairy and fruit juice processing [37], as well as for separating biopesticides from fermentation broths [38]. MF membranes are particularly valuable in wastewater treatment because they can recover nutritionally essential proteins and prevent residual iron and other harmful components from precipitating together with the proteins [39]. Ceramic MF membranes have also gained increasing attention for industrial wastewater treatment. Unlike polymeric membranes, ceramic membranes have a main drawback: high cost [40]. This has led to investigations into the use of low-cost fly ash as a raw material for membrane fabrication. Fly ash can contaminate water and, when inhaled, may damage vital organs such as the lungs, heart, and brain; therefore, its use in membrane production is considered a sustainable alternative [41].

UF is a pressure-driven membrane separation technology operating between MF and NF, with applications in drinking water treatment and biopharmaceutical processing [42]. UF separates solutes based on molecular size and effectively removes suspended particles from wastewater, producing high-quality water suitable for reuse while significantly reducing microbial loads by retaining viruses, colloids, and suspended solids [43]. UF membranes typically have pore sizes between 1 and 100 nm, allowing water, salts, and small organic molecules to pass while rejecting larger molecules and particulate matter [44]. Effluent turbidity is usually below 0.1 NTU, and particle removal efficiencies can reach 99.9%, which explains the widespread adoption of UF for its stable effluent quality and superior turbidity reduction compared to conventional systems [45]. UF-treated water often meets microbial safety standards without additional disinfection, reducing chemical dosing and enabling higher operational automation. In addition, UF systems can achieve up to a 5-fold reduction in water and energy consumption compared with many conventional and advanced treatment technologies [46]. Compared with MF, UF removes smaller particle fractions and achieves higher removal efficiencies for viruses and organic contaminants, although at higher operating pressures [47,48]. UF membranes are increasingly integrated with advanced oxidation, biological treatment, or other membrane processes such as forward osmosis to enhance overall performance [49], while current development trends focus on energy efficiency, cost reduction, and antifouling improvements [50]. In urban stormwater and snowmelt treatment, UF membranes effectively remove suspended solids, oil fractions, microorganisms, and colloidal contaminants. Case studies report reductions in total suspended solids and turbidity to below-detectable levels, alongside substantial decreases in total organic carbon and selected dissolved metals, thereby enabling compliance with international standards for non-potable reuse, such as irrigation and street cleaning [51].

Forward osmosis (FO) follows the natural osmotic process in which water molecules migrate from one solution to another through a semipermeable membrane. In FO, a highly concentrated draw solution (DS) is used to establish the concentration gradient that pulls water from the feed solution (FS). This gradient generates the osmotic pressure difference required to drive water transport from the FS toward the DS, and the process continues until chemical potential equilibrium is reached [52]. FO has been applied to treat and concentrate various wastewater streams.

Membrane bioreactors (MBRs) integrate biological degradation with membrane-based separation, enabling the production of consistently high-quality treated water within compact and modular systems [53]. High effluent quality results from the effective removal of suspended solids and pathogens, while elevated biomass concentrations reduce system footprint and support flexible adaptation to variable wastewater volumes and loads, making MBRs suitable for decentralized and scalable urban applications [54]. MBRs have gained increasing attention for their ability to remove antibiotics, addressing concerns related to antibiotic resistance and environmental and human health protection. In contrast to conventional activated sludge systems, MBRs demonstrate strong removal potential, with sponge-based configurations achieving complete tetracycline removal and 93–99% removal of norfloxacin, while flat-sheet membranes outperform hollow-fibre designs in antibiotic elimination [55,56,57]. Recent MBR innovations further enhance system flexibility and applicability. Designs such as the AirLift sidestream MBR and Direct Sludge Filtration enable high-flux operation with reduced footprint and simplified installation by separating biological and membrane compartments. Their plug-and-play configuration supports decentralized deployment and cost-effective retrofitting of existing treatment plants, contributing to adaptive urban wastewater treatment infrastructures [58].

Membrane photobioreactors (MPBRs), in contrast to conventional MBRs, integrate photosynthetic organisms such as algae or photosynthetic bacteria, which utilise light to drive their metabolic functions [59]. The membrane in MPBRs performs similar roles as in standard MBRs, including biomass concentration and separation [60]. The key distinction lies in the reliance on light and the cultivation of photosynthetic organisms, which differentiates MPBR systems from the more commonly used MBRs in wastewater treatment [61]. When supported by adequate pretreatment, such as activated sludge, MPBRs can achieve more than 50% removal of nitrogen and phosphorus from wastewater [62] (Table 1).

##### Case Studies of Water Reuse Projects Using Membrane Systems

Case studies consistently demonstrate that membrane technologies enable high-quality urban water reuse across diverse sources, including groundwater, municipal wastewater, rainwater, cooling tower blowdown, and decentralized building-scale systems. Reverse osmosis (RO) is particularly effective where near-drinking-water quality is required, achieving near-complete removal of inorganic contaminants (e.g., Mn, As, NH_4^+^_), dissolved organic matter, and humic substances, although with 8–10% higher treatment costs, partial boron removal, and the need for careful pretreatment to mitigate fouling and oxidation [63]. In industrial and cooling applications, RO performance is strongly dependent on pretreatment, with UF and MF effectively reducing turbidity, SDI, and iron content to improve operational stability [64].

Low-pressure membrane systems play a central role in decentralized reuse. Ultrafiltration (UF) consistently outperforms microfiltration in direct municipal wastewater filtration, maintaining stable operation for 1–2 months compared to only a few hours for MF, due to slower fouling dominated by cake-layer formation; organic matter accounts for >70% of irreversible fouling, and physical cleaning alone remains insufficient for full flux recovery [65]. Gravity-driven UF systems treating rainwater and stormwater achieve reliable turbidity reduction below reuse thresholds using 0.02 μm membranes, with additional sand and activated carbon filtration improving COD and enabling low-energy, infrastructure-light reuse for irrigation and disinfection [66].

Advanced and hybrid membrane configurations further expand the potential for reuse. Forward osmosis (FO) offers low-pressure operation and improved fouling reversibility, particularly for highly concentrated streams, although freshwater recovery depends on draw-solution chemistry: monovalent-ion draw solutes typically require RO regeneration, whereas multivalent or nanoparticle-based solutes can be recovered using UF or NF membranes [29,67,68]. Membrane bioreactors (MBRs) provide robust pathogen and micropollutant removal in decentralized contexts, with hybrid systems achieving 66–97% antibiotic removal when combined with ozonation [69] and 98.83% removal of particulate microplastics (57.65% for fibrous particles) in municipal effluents [70]. At the building scale, AeMBR and AnMBR systems meet required microbial log-reduction targets, with life-cycle assessments showing the lowest environmental and health impacts for graywater-based systems meeting 100% onsite non-potable demand, despite additional collection infrastructure requirements [71].

Circular economy approaches further enhance sustainability, as end-of-life RO membranes can be upcycled into UF-like and NF-like membranes for gravity-driven tertiary treatment, achieving stable flux, regulatory compliance [72], and low energy demand while reducing waste and treatment costs [73,74].

#### 3.2.3. Membrane-Based Cooling Systems for Urban Heat Mitigation

Membrane-based semi-direct evaporative cooling (MSDEC) represents a promising low-energy alternative for building air-conditioning, as it uses the latent heat of water evaporation to provide sensible cooling while relying only on pumps and fans as active components [75]. Membrane-based evaporative cooling systems operate by transferring water vapor through a selective polymer hollow-fiber membrane, enabling sensible cooling while preventing liquid droplet carryover and microbial contamination [76]. The large specific surface area of the fibers enhances coupled heat and mass transfer, improving cooling efficiency compared to conventional DEC and IEC units [77]. In semi-direct configurations, internal baffles guide airflow along the membrane bundles, intensifying mixing and increasing wet-bulb effectiveness [78]. Since only pumps and fans are required as active components, these systems offer a low-energy alternative for building air treatment [75]. Membrane-based semi-direct evaporative cooling (MSDEC) combines membrane selectivity with evaporative cooling to deliver low-energy air conditioning for buildings. Polymer hollow-fiber membranes allow only water vapor transfer, preventing droplet carryover and reducing microbial risks, while their high specific surface area enhances coupled heat and mass transfer. Internal baffles improve airflow distribution and can increase wet-bulb effectiveness by up to 32% without excessive pressure losses when fiber geometry and airflow are optimized. These features make MSDEC systems suitable for energy-efficient indoor air treatment with high cooling performance and improved air quality [78].

Membrane-based cooling and dehumidification systems can substantially reduce building energy demand compared to conventional vapor-compression air conditioning. Englart and Rajski analysed a membrane-based fresh-air system combining a 35% LiCl liquid desiccant dehumidifier with a membrane evaporative cooler using hollow-fiber modules. Under summer conditions, the system achieved a 9.7 °C reduction in supply air temperature while maintaining indoor comfort. Most processes were driven by low-grade thermal energy, with electricity consumption limited to fans and pumps. Solar-assisted desiccant regeneration and membrane-based heat and mass transfer enabled a coefficient of performance of up to 33.2, significantly reducing the cooling load of conventional compressor-based systems [79].

Conventional vapor-compression air-conditioning systems become highly energy-intensive under high latent heat loads, often leading to elevated indoor humidity and reduced air quality. In contrast, heat-driven, membrane-assisted liquid desiccant cooling systems provide effective dehumidification while significantly reducing cooling energy demand. Ghosh et al. demonstrated that an indirect-contact system using a lithium chloride solution behind PVDF membranes performs particularly well in humidity-intensive applications such as hospitals and server rooms. The membrane barrier prevents desiccant carryover, reduces reliance on compression cooling, and enables integration with renewable or waste-heat sources, thereby supporting urban climate resilience and lowering peak electricity demand [80].

The combination of ceramic microfiltration (CMF) and Ca(OH)_2_-based softening provides an effective pretreatment strategy for cooling systems affected by water hardness. Experiments with treated wastewater showed that Ca(OH)_2_ dosing promoted CaCO_3_ precipitation, significantly reducing Ca^2+^ concentrations and scale formation. CMF membranes maintained stable flux without scaling, even in the presence of organic matter, confirming the suitability of membrane-assisted softening for cooling applications using recycled water in urban environments [81].

Kim et al. investigated a membrane-based evaporative cooling system using a hydrophobic, porous PTFE flat membrane that separates a water reservoir from an air channel while permitting water vapor transport. A coupled heat and mass transfer model showed that cooling performance depends on the mass-transfer coefficient, airflow rate, and channel geometry, with good agreement between predicted and experimental results. Bench-scale tests with parallel flat-membrane modules achieved substantial temperature reductions, with a characteristic COP of approximately 1.58 at maximum blower power. The hydrophobic membrane enables large evaporative surface areas without water leakage and allows integration into concepts such as the Maisotsenko cycle; however, increased air humidity and water demand indicate that long-term indoor use requires coupling with additional heat exchange or dehumidification [82].

Air-gap membrane distillation (AGMD) is a thermally driven membrane separation technique that uses temperature-induced vapor transport across a hydrophobic membrane and can be coupled with low-grade heat sources such as waste heat or solar thermal energy [83]. This configuration reduces heat loss and improves energy efficiency, making it suitable for integration into low-temperature renewable energy systems relevant for cooling and energy recovery applications [84]. Recent studies identify AGMD as a promising option for low-temperature, renewable-energy-driven cooling systems. Experiments with an H_2_O/LiBr working mixture show that desorption performance is mainly controlled by the temperature difference across the membrane, with higher gradients yielding increased vapor flux and cooling efficiency. Stable operation was achieved with fluxes up to 5.69 kg/m^2^ and without significant heat losses. Because the process can be driven by low-grade heat sources (75–95 °C), AGMD can be readily integrated with solar thermal or waste-heat recovery systems. Simulation results indicate that AGMD-based disrobers can serve as sustainable, decentralized components of future urban cooling systems [85].

#### 3.2.4. Green Infrastructure and Nature-Based Solutions

**Figure 6:**
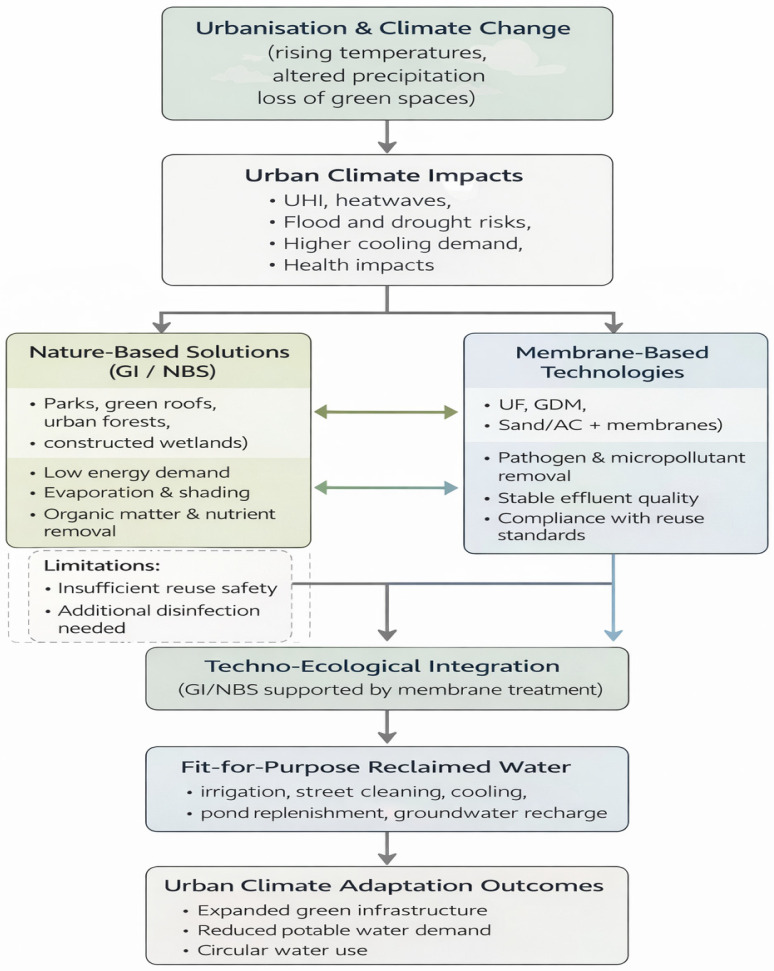
Techno-ecological integration of nature-based solutions and membrane technologies for urban climate adaptation and water reuse.

Urbanization and climate change jointly intensify risks related to rising air temperatures and altered precipitation patterns, as urban development and the loss of green spaces amplify climate impacts [86,87,88]. The reduction in vegetated areas contributes directly to the urban heat island (UHI) effect, leading to more frequent and intense heatwaves, increased cooling energy demand, higher air pollutant emissions, and adverse human health outcomes, as urban surfaces absorb and re-emit solar radiation more strongly than rural landscapes [89,90]. These impacts are exacerbated by the relative scarcity of vegetation and open water surfaces in cities, making urban environments particularly vulnerable to climate change [89]. In this context, green infrastructure (GI) and nature-based solutions (NBS)—including parks, green roofs, urban forests, and blue–green systems—play a central role in climate adaptation by regulating surface energy flows through evaporation, shading, and surface emissivity, while improving microclimate conditions, mitigating flood and heat risks, and supporting public health [91,92,93]. However, while NBS significantly enhance water security and climate resilience, they are often insufficient to ensure drinking-water safety without support from engineered systems [94]. This limitation has driven the emergence of techno-ecological approaches that integrate green infrastructure with built water treatment technologies, such as biofiltration and membrane systems, within urban water supply and reuse frameworks [95,96]. Biofiltration demonstrates how engineered systems can exploit natural microbial processes with relatively low energy and waste requirements [97]. The environmental performance of these hybrid systems can be assessed using criteria such as resource reliance, energy consumption, waste generation, and physical footprint, enabling comparison between nature-based and technology-assisted solutions [98]. Practical concepts such as IU-WA-RE demonstrate how treated municipal or industrial wastewater can supply green infrastructure with fit-for-purpose reclaimed water for irrigation and street cleaning, allowing the expansion of urban green spaces without increasing potable water demand, even in water-scarce regions [99]. Although regulatory uncertainty still constrains wider application in drinking-water contexts [100], techno-ecological systems provide a critical reference for developing integrated urban water strategies that link natural capital with engineered solutions and support long-term climate adaptation and water security (Figure 6) [101,102].

NBS have also been investigated for greywater treatment, mainly due to their inherently low energy demand and their effective capability for organic matter removal [103]. Constructed wetlands (CWs) are considered the most established NBS options for greywater management, while other nature-based approaches, such as green roofs and green walls, can similarly replicate key treatment processes characteristic of conventional CW systems [104]. Within CWs, biological degradation processes act together with physicochemical mechanisms, including precipitation, filtration, and adsorption, collectively enhancing organic matter removal efficiency [105].

For instance, Collivignarelli et al. [106] reported a chemical oxygen demand (COD) reduction of up to 89% during greywater treatment using CWs (Constructed Wetlands). Vegetated wetland systems generally achieve higher COD removal than unplanted configurations, as oxygen release in the root zone promotes aerobic degradation processes [107] and supports nutrient uptake by plants. Consequently, CWs may also function effectively as a polishing step following biological greywater treatment [108].

Constructed wetlands have furthermore demonstrated a strong capacity for nutrient removal from greywater streams [109]. Wetland vegetation facilitates the reduction of nitrogen and phosphorus concentrations, as these nutrients can be adsorbed and assimilated within the plant root systems [105]. High removal efficiencies for faecal coliform bacteria have also been documented in CWs, typically ranging between 94% and 98% [110]. Nevertheless, effluents treated by CWs usually require an additional disinfection step in order to comply with water reuse standards [106].

One study showed that sodium dodecyl sulphate (SDS), a commonly occurring anionic surfactant in greywater, can be removed with an efficiency of approximately 94% using CWs alone [111], outperforming removal rates reported for membrane bioreactor (MBR) systems [105]. In addition, both the support media and plant litter within wetland systems can undergo degradation over time [112], while the quality of the treated effluent can be maintained through appropriate control of hydraulic retention time [112].

Ultrafiltration membranes can act as an effective technical complement to urban stormwater management and nature-based solutions when polluted snowmelt or stormwater requires treatment. In the system studied by Kaykhaii et al., simple physical pretreatment followed by filtration with a hydrophilic PES/PVP UF membrane reduced suspended solids, turbidity, oil fractions, coliform bacteria, organic matter, and several dissolved metals to very low or non-detectable levels. The permeate met international and national standards for non-potable urban reuse, including street irrigation and the maintenance of wetland habitats, demonstrating that UF membranes can both reduce pollutant loads to receiving waters and support circular water use in urban green infrastructure systems [51].

Integrating membrane-based rainwater treatment into urban green infrastructure can enhance the performance and resilience of nature-based solutions. Gravity-driven membrane filtration (GDM) [73] offers a low-energy pathway to supply clean water for park irrigation and cooling applications by reliably reducing turbidity and stabilizing key quality parameters [113]. Complementary Sand/AC filtration provides additional removal of organic compounds and nutrients, supporting multi-purpose reuse within green spaces [114]. The combination of these systems enables decentralized production of “City Water” suitable for irrigation, spray cooling, pond replenishment, and groundwater recharge. Such membrane-supported treatment units can strengthen Sponge City strategies by ensuring consistent, quality-controlled water for diverse urban ecological functions [66].

#### 3.2.5. Decentralized Wastewater Treatment

Decentralized wastewater treatment provides a less resource-intensive and more ecologically sustainable alternative to centralized systems, while enabling water reuse and nutrient recovery in urban environments. Although small-scale treatment technologies are now capable of producing high-quality effluent, centralized infrastructure remains preferred due to perceived reliability concerns. When integrated into decentralized schemes, modern membrane technologies offer a robust treatment barrier that improves system performance and reduces reuse-related risks. Their modularity enables flexible application from individual buildings to cluster-scale systems, supporting adaptive urban water management and highlighting the relevance of membrane-based solutions for addressing remaining technical and institutional barriers in decentralized sanitation [115].

Recent empirical evidence indicates that the role of decentralized wastewater treatment in urban systems is highly context-dependent. Examples from peri-urban areas in Sweden show that such systems are primarily implemented where infrastructural or geographical constraints limit connection to centralized sewer networks. Adoption is not determined solely by income level, but also by factors such as infrastructure feasibility, flexibility requirements, environmental pressures, and local decision-making and sustainability objectives. These findings suggest that decentralized solutions function as adaptive, site-specific complements to centralised wastewater management rather than as uniform alternatives [116].

Membrane bioreactors are well-suited for decentralized greywater treatment due to their small footprint and their ability to cope with variable loadings and influent flow rates. Membrane filtration replaces gravitational settling, enabling the systems to deliver high effluent quality with a small footprint, typically achieving more than 96% COD removal and low nutrient and pathogen concentrations [117]. Microfiltration (MF), ultrafiltration (UF), nanofiltration (NF), and reverse osmosis (RO) membranes can be selected based on the target contaminants to be removed. However, a major limitation of membrane-based processes is concentrate generation, as contaminants are retained rather than eliminated [118]. MF membranes effectively remove particulate matter, producing permeate suitable for direct reuse, but show limited rejection of dissolved organic compounds, which may require post-treatment [119]. This limitation is mitigated in membrane bioreactors (MBRs), where biological processes enable the removal of smaller organic compounds.

Anaerobic membrane bioreactors (AnMBRs) are also being investigated for decentralized applications, as the combination of an anaerobic bioreactor and membrane filtration enables a substantial reduction in energy demand by eliminating aeration while allowing the recovery of renewable energy in the form of methane [120]. Based on pilot- and full-scale data, AnMBRs in decentralized systems achieve average removal efficiencies of approximately 99% for TSS and 89% for COD, while their energy demand is around 0.35 kWh/m^3^, which is substantially lower than that of aerobic MBRs (~3.6 kWh/m^3^). Methane recovery is estimated to enable electricity generation of approximately 0.32 kWh/m^3^, reinforcing energy efficiency and circular economy principles [121]. However, the application of AnMBRs for decentralized greywater treatment is still at an early stage, and therefore, only limited evidence is currently available to assess their performance under decentralized conditions [120].

Constructed wetlands (CWs) are engineered, nature-based wastewater treatment systems that rely on the combined action of plants, microorganisms, and substrate media. They are particularly suitable for decentralized wastewater treatment due to their low capital and operating costs, simple operation, and good tolerance to variable loadings. In the long term, CWs provide reliable removal of suspended solids and organic matter, typically exceeding 97%, while ammonia removal ranges from 70–86% and total nitrogen removal from 60–70%. Land requirements are relatively low in warm climates (1.5–2 m^2^/P.E.) but increase to 5–12 m^2^/P.E. in colder regions, with operating costs of approximately 0.1 €/m^3^ of treated wastewater [122]. CWs have been widely applied in small to medium-sized settlements, particularly in warm climates [123], but well-designed systems have also demonstrated effective performance in northern European countries such as Poland, Estonia, and Lithuania [122]. These characteristics make CWs a suitable option for decentralized applications, especially in areas with warm temperatures and sufficient land availability.

## 4. Discussion

### 4.1. Environmental, Technical, Economic, and Governance Constraints of Membrane Technologies

**Figure 7:**
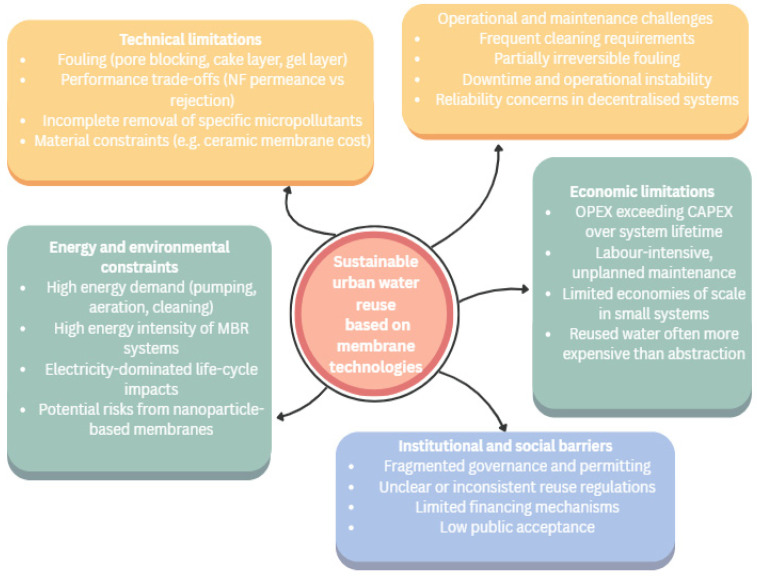
Interacting limiting factors affecting the sustainability of membrane technologies for urban water reuse.

Membrane technologies play a central role in urban water reuse and climate adaptation. Still, their large-scale and long-term application is constrained by interacting technical, environmental, economic, and institutional factors. Across membrane types, operational stability, fouling control, energy demand, and governance conditions emerge as decisive determinants of sustainability (Figure 7).

From a technical perspective, different membrane processes face distinct limitations. Nanofiltration (NF) systems may be associated with treatment-plant odour issues that affect operational conditions, while their performance is constrained by the inherent trade-off between water permeance and solute rejection [17,124]. Reverse osmosis (RO) is highly effective for removing salt and macromolecules. Still, it is energy-intensive, particularly for high-salinity feeds, due to the need for high operating pressures [125]. RO membranes are also vulnerable to fouling by salts, biological contaminants, and particulates, which increases cleaning frequency, downtime, and operating costs [125]. In addition, specific micropollutants, including pesticides, solvents, and PFAS compounds, are not entirely removed by RO, highlighting the need for complementary or alternative solutions [126]. Recycling and reuse of spent membrane modules has therefore been proposed as a partial mitigation strategy to reduce material and environmental burdens [127].

Forward osmosis (FO) offers advantages related to lower hydraulic pressure requirements, but its application is limited when the draw solution is not part of the final product. In such cases, additional separation steps are required to recover fresh water, increasing system complexity and energy demand. FO performance is further constrained by concentration polarisation effects that reduce effective osmotic pressure and permeate flux. When draw-solution regeneration relies on high-pressure processes such as RO, overall energy consumption can increase substantially [128].

Across membrane technologies, fouling remains one of the most critical challenges affecting long-term performance and cost-efficiency. Fouling reduces permeability, increases energy demand, and shortens membrane lifespan, thereby driving higher operational costs. Its development depends on membrane surface properties—including roughness, hydrophobicity, pore-size distribution, and surface charge—as well as feed-water characteristics such as pH and organic matter content [129]. Organic matter, inorganic salts, colloids, and microorganisms accumulate on membrane surfaces or within pores, causing pore blocking, cake-layer formation, and gel-layer development [130]. Rough or hydrophobic membranes are particularly susceptible due to enhanced particle adhesion [131]. Although backwashing, air scouring, and chemical cleaning can partially control fouling, these measures increase costs and environmental impacts and do not fully prevent long-term performance decline [132]. Consequently, the development of fouling-resistant membranes with smoother or more hydrophilic surfaces has become a key strategy to improve operational stability [133].

Experimental and pilot-scale studies confirm the central role of fouling and maintenance in determining membrane performance. Ceramic microfiltration experiments showed that sodium alginate induces severe flux decline through gel-layer formation and pore constriction, an effect intensified by Ca^2+^ complexation. Although Ca(OH)_2_ dosing partially removed calcium, it did not substantially improve flux behaviour, and flux recovery rates of 74–80% indicated partially irreversible organic fouling and limited cleaning effectiveness [81]. Similarly, decentralised rainwater-treatment pilots demonstrated that gravity-driven membrane filtration can achieve effective turbidity reduction with minimal energy demand. At the same time, long-term performance depends on filter-cake development and maintenance strategies [66].

Energy demand is a further limiting factor for membrane-based systems. Data from urban wastewater treatment plants in Beijing show that energy consumption is dominated by pumping, aeration, mixing, sludge recycling, and membrane-based advanced treatment. Aeration alone can account for more than one-third of total energy demand, while membrane filtration and cleaning further increase consumption. Comparisons among AAO, SBR, UF, and MBR systems indicate that MBRs exhibit higher energy intensities (0.57–0.725 kWh/m^3^), and increased plant capacity does not necessarily result in scale efficiencies [134]. Life-cycle assessments of decentralised greywater treatment further show that electricity use dominates the environmental impacts of community- and neighbourhood-scale MBRs. In contrast, household-scale systems perform poorly due to the lack of economies of scale [135].

Economic analyses highlight that operational expenditure often exceeds capital costs over the system lifetime. In UK drinking-water membrane plants, life-cycle operational costs were three to six times higher than capital expenditure over 20 years, largely due to labour-intensive, unplanned maintenance associated with membrane damage, clogging, and permeability loss. Approximately half of the total labour costs were linked to corrective interventions, particularly in systems below 30,000 m^3^/day capacity. Frequent or ineffective CIP further increased energy and chemical consumption while reducing membrane lifespan [136]. In stormwater treatment, UF systems deliver high-quality water but remain economically constrained by fouling-related maintenance and cleaning costs, despite performance improvements achieved through pulsatile flow operation [51].

To mitigate fouling, a wide range of surface-modification and material-based strategies has been developed. Increasing membrane hydrophilicity using PEG, PVP, or iron oxide nanoparticles reduces foulant adhesion [131]. Nanoparticle-based coatings such as TiO_2_, SiO_2_, and Ag provide antibacterial and photocatalytic properties, while carbon-based nanomaterials, including functionalised carbon nanotubes and graphene oxide, enhance hydrophilicity and reduce protein adsorption [137]. Additional approaches, such as UV photographing, organosilane functionalisation, smoother membrane surfaces, increased negative surface charge, and MOF fillers, further improve resistance to fouling and pore-structure stability [138,139,140].

Integrated assessments show that UF–RO systems achieve high pollutant-removal efficiency but are strongly influenced by fouling driven by dissolved organic matter, particularly hydrophobic and low-molecular-weight fractions. While RO can remove over 80–90% of DOM, severe fouling leads to flux decline and increased chemical cleaning, adding environmental and economic burdens [113].

Fouling mitigation through pretreatment and surface modification can reduce scaling and organic adsorption, but nanoparticle-functionalised membranes may introduce environmental risks related to leaching and long-term accumulation, requiring additional monitoring and regulation [141,142].

At the policy level, regulatory and institutional conditions strongly influence large-scale implementation. In the EU, the revised Urban Waste Water Treatment Directive aims for energy-neutral operation of plants above 10,000 p.e. by 2040/2045, partly through renewable energy integration [143].

However, reused water often remains more expensive than conventional abstraction, as illustrated by Italian cost comparisons and industrial reuse cases in Spain [144,145]. Sewer-mining pilots, such as the Athens case, demonstrate that MBR–UV systems can deliver unrestricted irrigation-quality water at moderate cost, while additional RO polishing is justified only when higher treatment levels are required [146].

Institutional fragmentation, unclear reuse guidelines, limited financing mechanisms, and low public acceptance further slow the expansion of wastewater reuse projects across Europe [26]. These barriers highlight that membrane technologies can contribute to urban water resilience only when technical performance, energy demand, life-cycle costs, regulatory compliance, and social acceptance are addressed in an integrated manner [144].

### 4.2. Integrating Membrane Technologies into Urban Resilience Strategies

Decentralised membrane systems play a strategic role in strengthening urban resilience by reducing reliance on centralised infrastructure and increasing supply flexibility. Building-scale non-potable reuse systems based on MBRs support integrated urban water management by reducing potable water demand and energy-intensive centralised treatment, while offering opportunities for coupling with alternative water sources and thermal recovery [71]. Similarly, decentralised rainwater-treatment concepts, such as City Water Hubs, demonstrate how gravity-driven membrane filtration combined with Sand/AC systems can reliably supply water for irrigation, cooling, and groundwater recharge, even under dry conditions [113,147].

Governance frameworks strongly condition the success of such systems. The IMPEL survey revealed substantial differences among EU Member States in implementing the Industrial Emissions Directive and the Water Framework Directive, with fragmented permitting and weak institutional coordination hindering membrane-based reuse, particularly in Portugal and Italy. In contrast, more stable regulatory frameworks and integrated permitting practices in countries such as the Netherlands and Germany facilitate technology adoption [148]. Outside Europe, Beijing illustrates how membrane-based advanced treatment can enhance resilience when embedded in a multilayered governance framework that aligns policies, institutions, and economic incentives, thereby making reclaimed water a major urban water source [134].

Urban planning instruments further enable local implementation. Sabadell’s dual distribution network and mandatory greywater regulations demonstrate how long-term planning, regulatory support, and financing mechanisms can institutionalise non-potable reuse at the city scale [149]. Data-driven decision-support tools and multi-criteria assessment methods support stakeholder participation, transparency, and acceptance, ensuring that membrane-based solutions align with local needs and long-term adaptation objectives [150,151,152].

Finally, EU-funded projects such as LIFE GREEN SEWER illustrate how integrated membrane systems combining FO–RO, AnMBR, and ultrafiltration can reduce pathogens and emerging contaminants while lowering energy use and water losses. Pilot results from Spain confirm high contaminant removal efficiencies and compliance with EU reuse regulations, demonstrating the potential of advanced membrane-based systems to support safe and efficient urban water reuse when embedded in appropriate technical and governance frameworks [153].

## 5. Conclusions

The reviewed literature confirms that membrane technologies are central to addressing rising wastewater volumes and water scarcity in urban environments, particularly where safe water reuse is prioritized by international and European policy frameworks (SDG6, EU water reuse requirements). Because conventional biological treatment cannot fully remove many micropollutants, UF, NF, RO, and membrane bioreactors provide the high removal efficiencies required to support urban water reuse and water security. Beyond micropollutant removal, membrane technologies are increasingly applied due to their ability to ensure reliable pathogen control, stable effluent quality, and compact, robust operation suitable for urban and decentralized systems. Decentralized applications, including building-scale non-potable reuse and rainwater or greywater treatment, reduce pressure on centralized systems and enable more adaptive urban water management. In parallel, membrane-based cooling technologies offer lower-energy alternatives for mitigating urban heat stress and peak energy demand. Green infrastructure and nature-based solutions depend on reliable supplies of appropriately treated reclaimed water, which membrane systems can deliver in a controlled manner. However, long-term sustainability is constrained by fouling, cleaning demand, energy and chemical consumption, and operational costs, highlighting the need for integrated technical, environmental, and economic assessment.

While membrane-based systems offer significant potential for urban climate adaptation, their long-term sustainability is constrained by fouling, cleaning requirements, energy and chemical use, and associated operational costs. These challenges underscore the need for integrated technical, environmental, and economic assessment frameworks. At municipal and policy levels, effective implementation also depends on coherent permitting processes, institutional coordination, and data-driven planning. Practical examples and EU-funded projects show that membrane technologies can be viable components of urban climate adaptation when applied at appropriate scales and with careful design. Future research should therefore prioritize fouling-resistant membranes, integrated life-cycle and risk assessment approaches, and solutions compatible with climate-adaptive hybrid urban systems.

## Figures and Tables

**Table 1:** Membrane technologies and their application in the urban environment.

Membrane Technology	Main Removal Targets	Typical Applications	Reported Advantages	References
**Reverse osmosis (RO)**	Dissolved minerals, salts, and organic substances	Drinking water production; industrial, residential, commercial and scientific water treatment	Produces high-quality water; widely applied; relatively low cost	[27,28]
**Nanofiltration (NF)**	Micropollutants, dyes, colour, selected monovalent ions	Brackish water treatment; industrial processes; tertiary wastewater treatment	Lower pressure than RO; higher permeate flux than RO; higher rejection than UF	[27,29,30,31,32,33,34]
**Microfiltration (MF)**	Suspended solids, microorganisms, oil fractions	Industrial wastewater; runoff water; dairy and fruit juice processing; fermentation broths	Enables recovery of proteins; prevents precipitation of harmful components	[36,37,38,39,40,41]
**Ultrafiltration (UF)**	Viruses, bacteria, colloids, suspended solids	Drinking water treatment; biopharmaceutical processing; urban stormwater and snowmelt treatment	Stable effluent quality; turbidity <0.1 NTU; high particle removal (up to 99.9%); reduced need for disinfection; high automation potential	[36,42,43,44,45,46,47,48,49,50,51]
**Forward osmosis (FO)**	Water separation from wastewater streams	Wastewater treatment and concentration	Operates without hydraulic pressure	[52]
**Membrane bioreactors (MBRs)**	Suspended solids, pathogens, and antibiotics	Decentralised and scalable urban wastewater treatment	High effluent quality; compact footprint; modular design; effective antibiotic removal	[53,54,55,56,57,58]
**Membrane photobioreactors (MPBRs)**	Nitrogen and phosphorus (>50%)	Wastewater treatment with photosynthetic systems	Combines membrane separation with nutrient removal	[59,60,61,62]

## Data Availability

The original contributions presented in this study are included in the article/Supplementary Materials. Further inquiries can be directed to the corresponding author.

## Supplementary Materials

The following supporting information can be downloaded at: https://www.mdpi.com/article/10.3390/membranes16020070/s1, Table S1: PRISMA 2020 Checklist [154].
